# Perceptual Learning with Complex Objects: A Comparison between Full-Practice Training and Memory Reactivation

**DOI:** 10.1523/ENEURO.0008-19.2021

**Published:** 2021-03-10

**Authors:** Chiu-Yueh Chen, Hans Op de Beeck

**Affiliations:** 1 Brain and Cognition, Katholieke Universiteit Leuven, Leuven 3000, Belgium; 2 Leuven Brain Institute, Katholieke Universiteit Leuven, Leuven 3000, Belgium

**Keywords:** consolidation, object recognition, perceptual learning

## Abstract

Perception improves with repeated exposure. Evidence has shown object recognition can be improved by training for multiple days in adults. Recently, a study of Amar-Halpert et al. (2017) has compared the learning effect of repetitive and brief, at-threshold training on a discrimination task and reported similar improvement in both groups. The finding is interpreted as evidence that memory reactivation benefits discrimination learning. This raises the question how this process might influence different perceptual tasks, including tasks with more complex visual stimuli. Here, this preregistered study investigates whether reactivation induces improvements in a visual object learning task that includes more complex visual stimuli. Participants were trained to recognize a set of objects during 5 d of training. After the initial training, a group was trained with repeated practice, the other a few near-threshold reactivation trials. In both groups, we found improved object recognition at brief exposure durations. Traditional intense training shows a daily improvement; however, the group with reactivation does not reach the same level of improvement. Our findings show that reactivation has a smaller effect relative to large amounts of practice.

## Significance Statement

Perceptual learning helps to explore adult plasticity in visual processing. Gradual improvements in the perception of complex objects have been demonstrated across multiple daily training sessions of hundreds of trials. These improvements in the trained objects and the transfer to new objects, in that sense, support “practice makes perfect.” Recent research challenges this idea, and suggests that a few critical reactivation trials can boost the learning processes. Here, we extend this idea to other learning tasks and investigate the extent to which short reactivation with a small number of trials can replace extensive training with complex visual objects. In our paradigm, we found larger training effects with extensive training.

## Introduction

Sensory information processing can be improved, but improvement requires repetitive practice. Repeated presentations of the same stimuli induce perceptual learning. A substantial body of evidence demonstrates training-induced improvements in the perception of relatively simple visual aspects, such as frequency discrimination (Fiorentini and Berardi, 1981), orientation discrimination (Aberg et al., 2009; Jeter et al., 2010), and feature discrimination (Karni and Sagi, 1993; Censor et al., 2006; Censor and Sagi, 2008; Amar-Halpert et al., 2017). Interestingly, similar learning curves have been reported with more complex objects (Furmanski and Engel, 2000; Baeck et al., 2012, 2014, 2016; Van Meel et al., 2016). This so-called object learning involves improved recognition of objects after multiple days of training. This improvement in object recognition under perceptually challenging conditions seems related to activity in high-level object-selective cortex (Grill-Spector et al., 2000; Van Meel et al., 2016).

While the boundary between simple and complex processing is difficult to draw, together these findings demonstrate that a wide variety of visual capabilities improve with extensive trainings. Recently however, in a texture discrimination task, the same profile of learning across days was observed using only a limited number of reactivation trials in the subsequent training days (Amar-Halpert et al., 2017). This study made use of a hypothetical window of opportunity during memory consolidation, where memories are re-evoked in the subsequent days after the initial encoding training. This reactivation needs only a few trials but results in similar behavioral improvements compared with the usual practice with hundreds of training trials. A similar mechanism in the domain of motor learning suggests memory consolidation because of reactivation in the context of a finger-tapping task (Walker et al., 2002, 2003). In that case, the induced performance benefits occurred in a brief reactivation which is <60 s (de Beukelaar et al., 2014). Although these training paradigms have shown stable and long-lasting effects in simple motor and visual learning with such short reactivations, no study has explored if the same phenomenon can be observed in more complex processing. The number of studies on this phenomenon is very limited and it is important to explore the necessary and sufficient conditions of these effects.

Another aspect to consider in visual learning is learning selectivity. Indeed, the training-induced learning effects are observed specifically for the stimuli set used for training. However, the degree of generalization *(*or transfer) of learning to new (but related) stimuli varies. In fact, the learning effects can show a lack of generalization to other objects (Furmanski and Engel, 2000). On the other hand, studies have typically noticed a partial generalization across stimulus size (Furmanski and Engel, 2000) and unseen images of the same objects (Baeck et al., 2016). It is unclear how the aforementioned reactivation protocol would interact with specificity and generalization.

This preregistered study will therefore test (1) whether the short reactivation strategy can induce object learning to the same extent as classical intensive training and (2) how selective this learning process is. The experimental design will in many details be based on earlier object learning experiments (Baeck et al., 2012), using the same materials and dependent variable they used. In addition, we will add a between-subject manipulation of reactivation training similar to the procedure from Amar-Halpert et al. (2017). Based on the previous success with this reactivation protocol, we predict similar learning curves in this protocol compared with the standard object training protocol with a much higher number of trials.

Earlier studies on object recognition training have started with a group of common objects as stimuli. With hundreds of repetitive trials, recognition can be improved by training. However, we hope to uncover the detailed reasons for changes in object recognition. Now, our study aims to bridge the gap between object learning paradigms that involve long exposure and shorter paradigms that rely on memory reactivation. This work will inform us about the mechanisms underlying learning-induced improvements in object recognition. The larger the extent that memory reactivation is involved in such improvements, the more improvement we expect to find after brief periods of training that are designed to reactivate memory.

## Materials and Methods

The approved Stage 1 protocol, the anonymized study data and digital materials can be found on the Open Science Framework (OSF) at https://osf.io/utx6n/.

### Participants

Fifty-two participants in this study were randomly assigned to two groups: 26 in the full-practice groups (aged 22.5 ± 5.2 years, six males and 20 females) and 26 in the short-reactivation group (aged 22.1 ± 4.3 years, two males and 24 females).

Participants of either gender (aged between 18 and 40) were recruited online through a university online recruitment system (SONA), Facebook, as well as through banners and leaflets. The volunteers received monetary rewards.

Initially, a planned sample of 50 was set from a power analysis over 0.90. Data collection was prone to no-shows, which is why we scheduled >50 participants. In the last week of testing, we needed one participant but scheduled three and all three showed up. As such a final sample of 52 was obtained.

There was no a priori limit on the proportion of male/female participants. The experiment was approved by the Social and Societal Ethics Committee of Katholieke Universiteit Leuven (G-2017 121045). Participants signed an informed consent before every session. The following criteria was used to exclude a participant’s data: (1) a participant does not attend all sessions; (2) the obtained threshold value on the first or the last day is worse than the baseline (>120 ms).

On top of the final sample of 52, three more participants started with the first session, but were removed from the data file; one because of the obtained threshold value on the first day (140.94 ms) was worse than the starting value of 120 ms set a priori, one because of cancelation of the later slots, and one because of being outside of the predefined age range.

### Apparatus

The whole experiment was conducted using a Dell desktop computer (GX-780), using MATLAB and Psychtoolbox 3 (Brainard, 1997; Pelli, 1997; Kleiner et al., 2007). Visual stimuli were displayed on a 16-inch CRT monitor (Dell 790) with a 1024 × 768 pixels resolution at 100 Hz. The room was dim and viewing distance was 90 cm.

### Stimuli

The full stimulus set consisted of 40 gray-scale pictures of common manmade and natural objects that were used in a previous study (Baeck et al., 2012). The contrast of stimuli was reduced to 12.5% of the original contrast to make objects harder to recognize. Masking stimuli are made by a combination of fragments (70 × 70 pixels) of all different object pictures. Image size was 450 × 450 pixels (8.7 visual degrees). All stimuli were γ corrected to create a linear luminescence range. As the γ correction decreased overall contrast, an inverse γ-correction was applied to the masking stimuli to increase the contrast of the masks and thus obtaining a more robust masking effect.

#### Selection of image set

In Beaeck et al.’s (2012) work, the large stimulus set was divided in two subsets. One subset was used for training the other subset as a control. This design assumed that 20 stimuli per subset would be sufficient to average out possible stimulus-specific variations in difficulty (e.g., a stimulus that is more difficult than other stimuli). However, the large number of stimuli results in less exposure to each individual stimulus. In the current study there is an additional disadvantage, namely that the number of reactivation trials goes up linearly with the number of stimuli. For that reason, we decided to limit the number of stimuli in each subset to five stimuli.

We partitioned the stimuli in six subsets of five stimuli, then tested five pilot participants with six two-down, one-up staircases per subset. The obtained thresholds of these pilot trials helped us to select a number of stimulus subsets with equal difficulty. However, the task with five stimuli became so easy that the responses were achieved at the minimum stimulus duration without fluctuation, making it impossible to investigate the main effect. To prevent visual adaptation, the stimulus size was changed randomly from 250 pixels to 450 pixels, and this additional need for size invariance increased the difficulty of the recognition at short stimulus durations.

**Figure 1.**
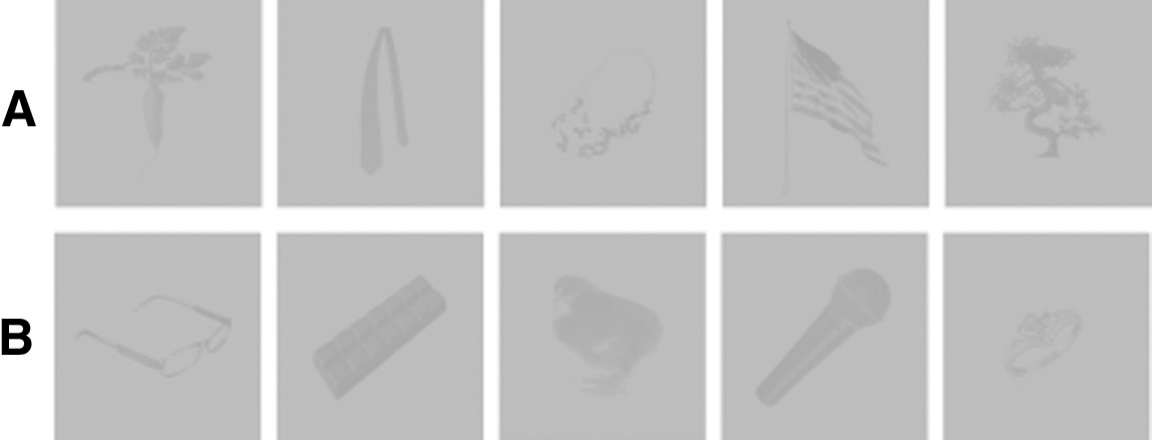
Two stimulus sets. Each set contains five different object categories. Panels ***A*** and ***B*** display 2 of the 6 subsets used in the experiment.

Two stimulus sets (Fig. 1) were counterbalanced across participants so that all stimulus sets will be included to the same extent as training and control stimuli between the two subject groups.

### Object learning task

**Figure 2.**
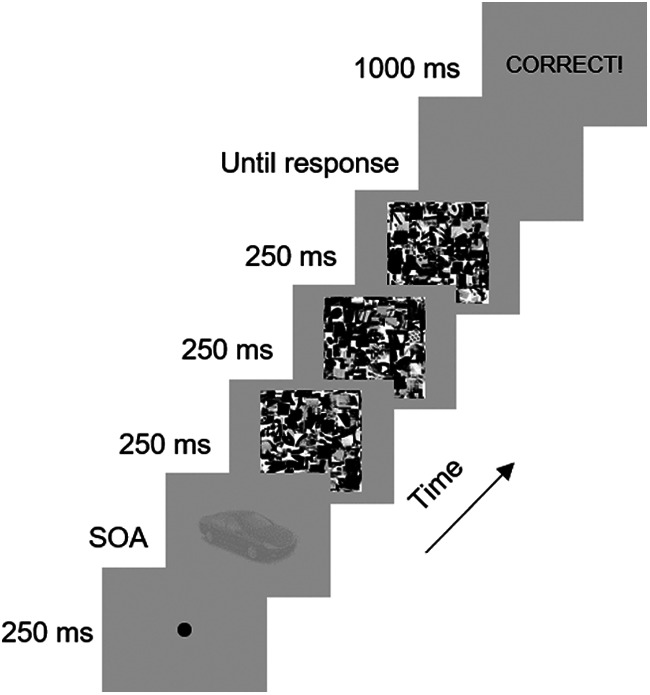
Example of one trial. The durations between the object and the mask stimuli (SOA) are determined based on an adaptive interleaved two-down, one-up staircase procedure. The object stimulus in this example trial is a car (“auto”). If the participant types “au,” the feedback “CORRECT!” (English: “CORRECT”) is shown. If the participant gives another response, then “FOUT! HET was auto” (English: “WRONG! It was car”) appears.

Each trial started with a fixation and the stimulus presentation for a variable time [stimulus onset asynchrony (SOA)]. Next, three consecutive masks were presented at the same location for 250 ms each, to prevent further visual processing (Op de Beeck et al., 2007). The presented position of stimuli and masks was randomized with a maximum deviation of 1.8 degrees from the screen center, the size-invariant stimuli and masks ranging randomly from 250 × 250 pixels to 450 × 450 pixels. The variable stimulus duration was determined through two interleaved two-down, one-up staircases. Stimulus duration was initially set at 120 ms (12 frames at a 100-Hz refresh rate), decreased by 10 ms (one frame) after two consecutive correct answers and increased by 10 ms after each incorrect answer. Participants were requested to type the first two letters of the name of the presented object. Three-letter responses have been used before (Baeck et al., 2012), but we can simplify this to two letters because stimulus sets included only five stimuli. A “true” or “false” feedback was shown after each trial. In case of a wrong answer, the correct object name was provided (see Fig. 2).

Note that the procedure of shortening stimulus duration as the SOA gets smaller in could arguably result in a situation in which challenges in recognition performance are because of reductions in perceived stimulus contrast in low-level visual areas. We cannot rule out this possibility, yet it is assuring that the same manipulation was used in previous studies that found correlations with object recognition performance in object-selective cortex (Grill-Spector et al., 2000) and causal effects after stimulation in lateral occipital cortex (Van Meel et al., 2016).

### Procedure

Participants were randomly assigned to two training paradigms (full-practice vs short-reactivation trainings). Each participant was trained with one subset of five stimuli. Threshold values are the average of the last four reversals of all staircases per day.

One other subset of five stimuli was used as a control set for individual participants, only to be seen during the first and last session. Across participants, each stimulus subset served equally often as a training set and as a control set.

The participants in the full-practice group completed standard training sessions between the first and the last day (see Table 1). The other participants in the short-reactivation group completed a standard training session on the first day and perform only short reactivation on the other 3 d. The fifth day is a test session which is the same for both groups.

**Table 1:** Study design

Full-practice training paradigm			
Time	Day 1	Days 2–4	Day 5
Session	First test and training	Training	Final test
Training trials	400	800	200
Control trials	400		200
Short-reactivation training paradigm			
Time	Day 1	Days 2–4	Day 5
Session	First test and training	Reactivation	Final test
Training trials	400	25	200
Control trials	400		200

Procedure of the adapting training sequence for the full-practice training paradigm and the short-reactivation training paradigm.

#### Preview

In earlier work (Furmanski and Engel, 2000; Baeck and Op de Beeck, 2010; Baeck et al., 2012, 2014, 2016; Van Meel et al., 2016), each session started with a preview of all stimuli presented in that session. A preview of the stimuli (2 s each) with their corresponding names was displayed to ensure that participants know the object images and their names. In the present experiment, we included this preview in the first and the last session for all 10 object stimuli (two subsets). The preview was not shown in the intermediate training sessions to not interfere with the reactivation procedure.

#### First test and training session

The first session involved a preview and eight experimental blocks of 100 trials (800 trials). Each block comprised two interleaved staircases of 50 trials. Baeck et al. (2012) only used 40 trials per staircase, but we expect to be able to collect more trials per unit of time because the required responses have been simplified (two instead of three letters). Each block included only five stimuli, either the stimuli that were trained or the control stimuli. Of the eight blocks in the first session, four blocks included trained and four blocks control stimuli, interleaved, and the stimulus set that came first was counterbalanced across participants. The total duration of the experimental session on day 1 would last 1 h.

#### Training session

A standard training session involved eight experimental blocks of 100 trials (800 trials) with each block comprising two interleaved staircases of 50 trials. Standard training sessions only included the trained stimuli, the control stimuli were not shown.

#### Reactivation session

Participants performed five near-threshold trials of each stimulus, resulting in 25 reactivation trials per session. Threshold values were the average of the last four reversals of the last four staircases on day 1 for that participant.

#### Final test session

The test session on the final day was the same as the first half of the first session, with a preview of the 10 object images and four blocks of 100 trials. This test session was shorter than the first session, because it only served to assess the thresholds and not to induce a large amount of training.

### Analysis pipeline

Learning-related changes were quantified by the threshold values across sessions, which is the most obvious manner to characterize performance when using an adaptive procedure (psychometric curve fitting is difficult given the uneven sampling of this function, see Baeck and Op de Beeck, 2010). For each participant, threshold values were the average of the last four reversals of every staircase complete with a stimulus set in a session. A lower threshold value corresponds to better performance. If the obtained threshold on day 1 or day 5 is worse than the baseline (120 ms), the individual data will be excluded. The data were presented as mean and the SEM of two stimulus sets (trained, control), two training paradigms (full-practice, short-reactivation) and time (sessions). A decline in thresholds over sessions indicates a training effect.

Before analyzing the training effect, individual data on the initial session was checked for equivalence between the two training paradigms. Next, the main analysis evaluated learning effect with *t* tests in line with Baeck and Op de Beeck (2010); Baeck et al. (2012, 2014, 2016) and Van Meel et al. (2016). In order to test the central hypothesis of the current paper, the final session performance in the two groups was compared with find out whether the full-practice group has reached lower thresholds compared with the short-reactivation group. We further tested the specificity for the control stimuli among different training groups. Results are reported with *p* values, confidence interval (CI), and effect size (see Table 2).

**Table 2:** Summary of statistical analysis

	Data structure	Type of test	Power/CIs
a	Normal distribution	Independent *t* test	0.98
b	Normal distribution	Independent *t* test	0.07
c	Normal distribution	Independent *t* test	0.09
d	Normal distribution	Repeated measures ANOVA	1
e	Normal distribution	Dependent *t* test	95% CI [12.35,17.80]
f	Normal distribution	Dependent *t* test	95% CI [8.78,14.63]
g	Normal distribution	Independent *t* test	0.73
h	Normal distribution	Independent *t* test	0.76
i	Normal distribution	Bootstrap *t* test	95% CI [−15.54,−2.70]
j	Normal distribution	Independent *t* test	0.67
k	Normal distribution	Dependent *t* test	95% CI [7.66,14.17]
l	Normal distribution	Dependent *t* test	95% CI [7.17,11.96]
m	Normal distribution	Independent *t* test	0.17
n	Normal distribution	Independent *t* test	95% CI [2.34,6.79]
o	Normal distribution	Independent *t* test	95% CI [−1.81,4.55]

#### Equivalence test for comparing the groups before training

To confirm that the observed training effects are meaningful when assessed from the performance in the last day, we evaluated whether the two groups had equal performance on the first session using an independent *t* test. We did not run an equivalence test as proposed originally because we did not set the bounds of the latter a priori. Irrespective of the outcome of this analysis, we also report the results when data were normalized for the performance on day 1. The same overall conclusions are reached with and without normalization, although quantitatively the numbers change.

#### Overall training effect

The main analysis assessed the effect of training between day 1 and day 5, with two paired *t* tests for each training paradigm.

#### Group comparison in terms of training effect

We compared the two groups in two ways. First, we compared the day 5 performance between the full-practice and the reactivation group with an unpaired *t* test. Second, as done by Amar-Halpert et al. (2017), we would estimate the level of improvement. The training effect is sometimes referred to as a learning rate. Here, to use a consistent terminology relative to the study of Amar-Halpert et al. (2017), we also use the term learning rate. The learning rate would be computed that divided the differences of day 1 and a target day by day 1 performance, for example (day 1 – day 5)/day 1, multiplied by 100 to obtain percentages.

#### Specificity of object learning

Following the analysis of the overall learning effect, we investigated the specificity of the training in days 2–4 to the untrained stimuli. Two paired *t* tests were conducted to compare the control stimuli performance between the first and final test sessions within a group of subjects. An unpaired *t* test was conducted to compare the day 5 performance between the full-practice and the short-reactivation group.

### Effect and sample size calculation

The key comparisons made by Amar-Halpert et al. (2017) in the 5-d standard practice, memory-reactivation, and 2-d standard practice suggest that the brief reactivations during training do improve discrimination thresholds. The original paper did not provide the raw and average values at the test and retest sessions; we estimated the pooled SD to be 20% within two groups from the given SEM of 5.9% and 5.5% in each group. Here, we present effect sizes and required sample sizes, calculated with a data analytics software (G*Power 3.1.9.2; RRID:SCR_013726).

Amar-Halpert et al. (2017) reported that learning rates in the two groups ranged from 20.6% (memory reactivation) to 26.6% (standard practice), with a nonsignificant difference (*F*_(1,22)_ = 0.56, *p* = 0.46) between total learning rates in the standard-practice and the memory-reactivation groups.

Despite a relatively low number of participants per group (*N* = 12), the study of Amar-Halpert et al. (2017) had a reasonable power to detect a learning effect in a group, because these effects are large. To find an effect of 20.6% with SD of 20%, the power is 0.75. If our null hypothesis is that memory-reactivation would result in no learning effect at all and the alternative hypothesis states that there is as much learning as in the standard practice group, then with *N* = 18 we would have a power of 0.90. However, we could also hypothesize that the reactivation might result in some learning, albeit much smaller than in the standard-practice group. Thus, to safeguard us against this possibility, we opt for a sample size of *N* = 25^a^ in each group, which is double the number in the original study.

## Results

All data and analysis scripts are publicly available on the OSF (https://osf.io/utx6n/). This study obtained 52 participants, 26 in the full-practice group and 26 in the short-reactivation group. Participants in both groups performed a 5-d training with the same amount of trials on the first and final days. Participants in the full-practice group performed a standard training session of 800 trials in three daily training sessions (days 2–4). Participants in the short-reactivation group performed 25 at-threshold trials in three daily reactivation sessions.

Threshold values, representing the performance per subject, are the average of the last four reversals of every staircase. The reversals include local maximums and local minimums. The local maximums are calculated from a function (findpeaks) in MATLAB. In order to perform this analysis of the local minimums, the data are multiplied by –1.

The initial performance on full-practice and short-reactivation training is tested for equivalence. Two-tailed *t* tests confirmed that both groups had similar performance on the first day. Using the data of both stimulus sets, there was no significant difference, *t*_(50)_ = 0.4569, *p* = 0.5793, *d* = 0.1267^b^, nor was there a difference when tested only on the thresholds for only the trained stimulus set, *t*_(50)_ = 0.6063, *p* = 0.5471, *d* = 0.1682^c^.

Although the difference is not significant, there is a trend toward higher initial thresholds in the reactivation group. Importantly, all our conclusions are not only backed up by day 5 performance, but also by analyses of the learning rates that take into account the baseline performance at day 1.

During the reactivation sessions, subjects were given brief training with the near-threshold trials. To estimate the individual threshold for each subject, the final four reversals of last four staircases on day one were averaged, mean ± SE: 40.75 ± 1.86 ms. Based on this, the stimulus duration used for the reactivation trials was 41.54 ± 2.05 ms (note that per participant the duration is a multiple of 10). The average accuracy during the reactivation trials was 60 ± 3.86% on day 2, 67.54 ± 4.03% on day 3, and 70.62 ± 4.29% on day 4.

### Learning effect

**Figure 3.**
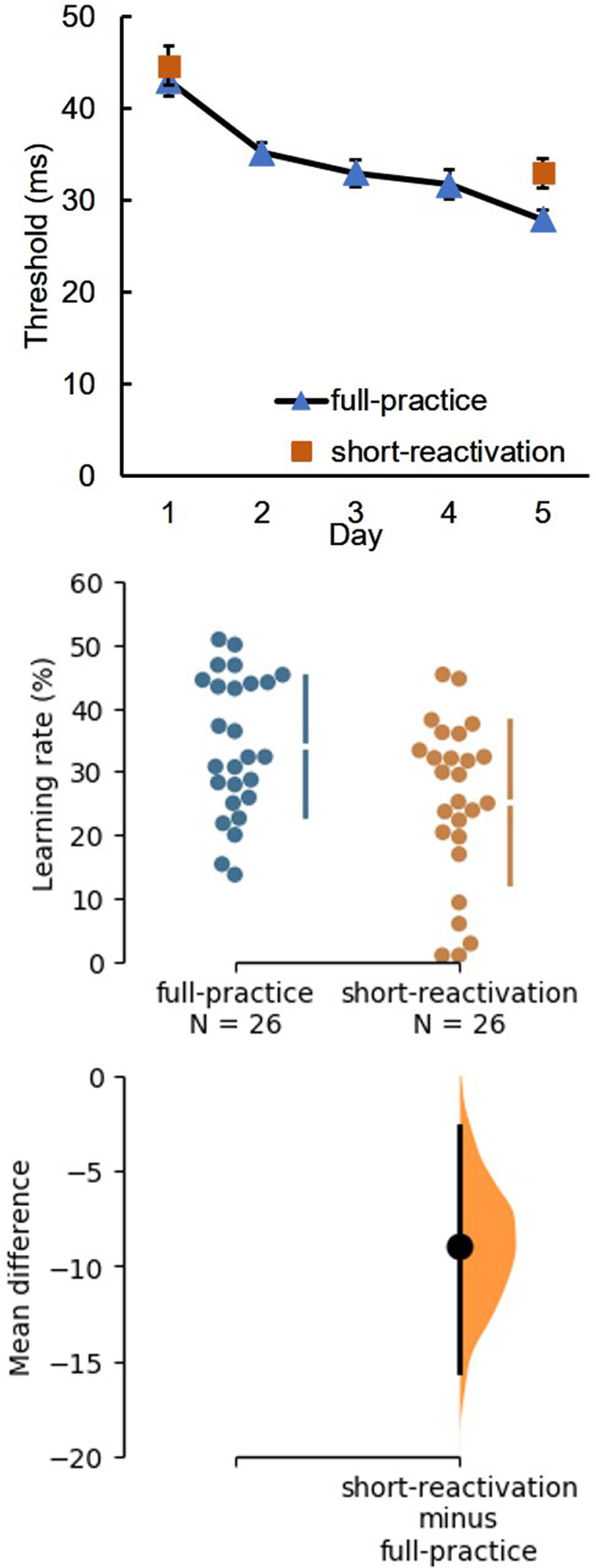
Overall learning effect in the full-practice group and the short-reactivation group for the trained stimuli. Top panel, Performance thresholds are plotted as a function of time and group. Error bars represent the SEM. Middle panel, The distribution shows the spread of the learning rates in each group. Circles represent the learning rates of individual participants. The gapped lines represent the SD of each group. Bottom panel, A bootstrapped resample distribution depicts the learning rate difference between two groups. A circle represents the difference between two groups of −8.90 and the end of the vertical black bars represent 95% confident interval of −15.53 and −2.70, *p* = 0.0072.

After testing the initial performance between two groups, the performance on trained stimuli after training was assessed with two paired *t* tests. Mean thresholds for both groups are shown in Figure 3. A learning effect was found for the full-practice group as in previous studies (*F*_(4,120)_ = 56.87, *p* < 0.001, η^2^ = 0.70^d^). Overall, performance thresholds were lower on day 5 than the day 1 in the full-practice group (*t*_(25)_ = 11.4, *p* < 0.001, *d* = 2.24^e^), as well as in the short-reactivation group (*t*_(25)_ = 8.24, *p* < 0.001, *d* = 1.62^f^).

Then we compared the learning-induced changes for the trained stimulus set between two groups, as inferred from the performance threshold on the final day and the learning rate. The a priori hypothesis was that participants in the short-reactivation group and in the full-practice group improved equally. However, participants in the short-reactivation group were found to perform less well on the final test session (day 5) than participants in the full-practice group, *t*_(50)_ = 2.6096, *p* = 0.0119, *d* = 0.72^g^. A similar finding was found when focusing on the learning rate. The learning rates for full-practice (Mean ± SD, 34.13 ± 10.93%) and for reactivation group (25.23 ± 12.74%) were significantly different (*t*_(50)_ = 2.7047, *p* = 0.0093, *d* = 0.75 h). For the key finding, we compared the mean difference of the learning rate between groups and performed a bootstrapped distribution by 5000 resamples. The difference of learning rates in two groups was 8.90% with a 95% CI from −15.54 to −2.70^i^.

Note that these comparisons still overestimate the effect of the reactivation sessions. Much of the improvement compared with day 1 might be a consequence of the many trials in day 1. Motivated by the surprising result of much smaller learning in the reactivation group, we performed an additional analysis that was not mentioned in the preregistration. We questioned whether there would be any beneficial effect of the reactivation trials if we take into account the training induced by day 1. We compared the learning rate at day 5 in the reactivation group, with the learning rate at day 2 in the full-practice group. These two cases are comparable in the sense that participants have received 1 day of training (day 1), with then the reactivation trials as an additional exposure for the reactivation group. This test is not free of confounds though, in particular, the time intervals are not the same, which should mostly bias us toward a null effect (no added benefit of the reactivation trials because potentially undone by a longer time interval). When tested, day 2 learning rate in the full-practice group (17.07 ± 11.14%) was lower than the day 5 learning rate in the reactivation-related changes, *t*_(50)_ = 2.46, *p* = 0.0175, *d* = 0.68^j^. This finding suggests that the reactivation had some beneficial effect, but, given our earlier tests, much less than a full practice protocol.

### Specificity

**Figure 4.**
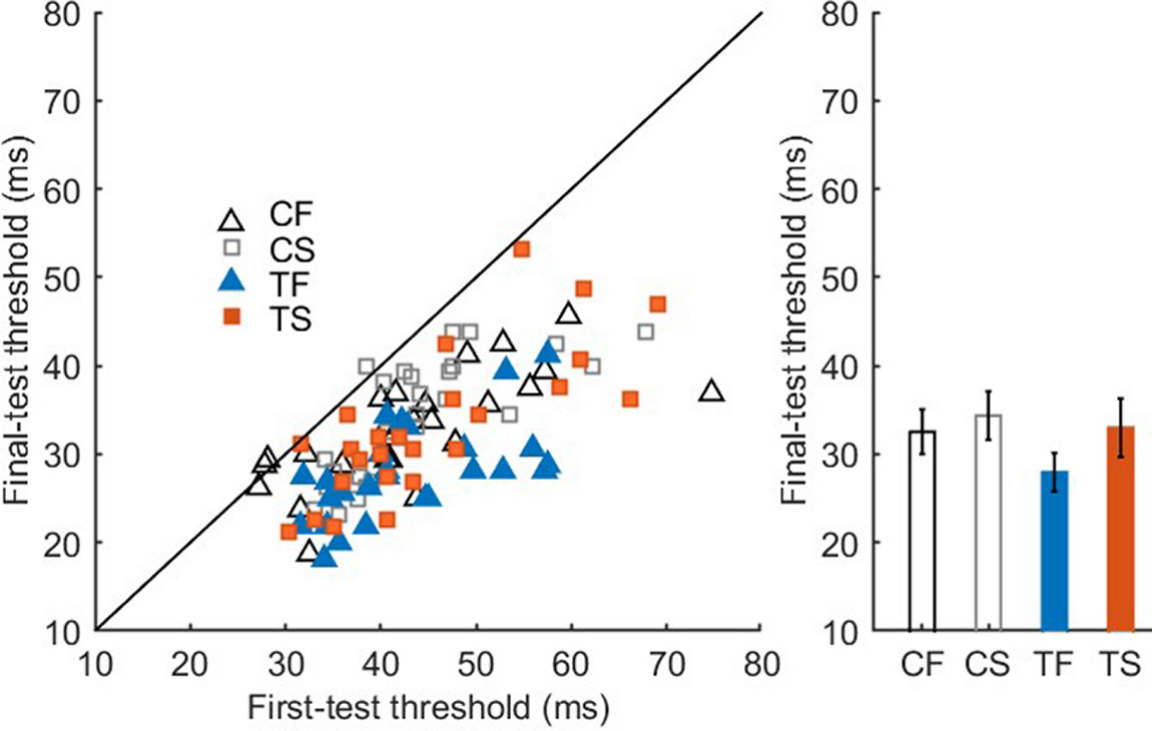
Recognition thresholds in the full-practice group and the short-reactivation group. Scatter plot of the thresholds of individual participants in the first and last day, plotted separately per stimulus set and per group (*N* = 26 in each group). The diagonal line represents the same performance on the first and last day, all points falling under this line represent a better performance on the last day compared with the first day. CF, control stimuli in full-practice group; CS, control stimuli in short-reactivation group; TF, trained stimuli in full-practice group; TS, trained stimuli in short-reactivation group. Error bars represent 95% CI.

Following the analysis of the learning effect, we investigated the specificity of learning to the trained stimulus set by analyzing the thresholds for the control stimuli that were only present at the first and final test sessions. The threshold for the control stimuli was lower on the final test session (day 5) than the first test session (day 1) in the full-practice group, *t*_(25)_ = 6.9011, *p* < 0.001, *d* = 1.35^k^, and in the short-reactivation group, *t*_(25)_ = 8.2303, *p* < 0.001, *d* = 1.61^l^ (Fig. 4). On the final test session, performance for the control stimuli did not differ between groups, *t*_(50)_ = 1.0095, *p* = 0.3176, *d* = 0.80^m^. Furthermore, on the final test session, participants in the full-practice group were better at recognizing trained stimuli than the control stimuli, *t*_(25)_ = 4.2261, *p* = 0.0, *d* = 0.83^n^, while this was not the case in the short-reactivation group, *t*_(25)_ = 0.8877, *p* = 0.3831, *d* = 0.17°.

## Discussion

The main objective of this study was to test the generality of previous work suggesting that in visual learning a short reactivation protocol results in as much learning as a more traditional time-intensive training. Our main finding is a significantly better learning effect in object recognition for participants who performed traditional repeated training. Although there is still a small beneficial effect of the reactivation protocol, this improvement is much less than what is achieved through a time-intensive training. The lack of strong training effects of the reactivation protocol with one set of stimuli is probably also the main reason why there is no specificity of training when comparing performance between the trained stimuli and another set of stimuli that were not shown during reactivation.

This study successfully establishes perceptual learning and extends the prior knowledge of the effects of memory reactivation. Our findings of a limited effect of memory reactivation resonate with other domains in which it has proven hard to identify the boundary conditions that influence the beneficial effects of memory reactivation. In several domains there has been a discussion that resulted from a variety of experimental outcomes. The original formulation of memory reconsolidation theory comes from the domain of fear conditioning. Researchers have hypothesized the stabled memory can be modulated and even destabilized by performing extinction training during the reconsolidation window (Monfils et al., 2009; Schiller et al., 2010). However, the effect of fear memory destabilization reported in the original studies could not always be replicated, sometimes the reactivation did not differ in or outside the hypothesized critical period, and in some studies there was even no effect of reactivation (Luyten and Beckers, 2017; Chalkia et al., 2020). Similar discussions have arisen in the domain of motor skill learning. Evaluated by obtaining a key typing task, new learning interfered with performance (Walker et al., 2003). The expected reconsolidation effect was absent in a direct and conceptual replication (Hardwicke et al., 2016; Walker and Stickgold, 2016). In the domain of perceptual learning, the positive findings of Amar-Halpert et al. (2017) are accompanied by reactivation effects in similar paradigms such as orientation discrimination (Bang et al., 2018). Overall, while a large literature also supports the effects of reactivation and its induced reconsolidation (for review, see Lee et al., 2017), a lot remains to be done to understand the boundary conditions under which a reactivation protocol is effective.

It is important to note that our study was not meant to directly replicate the study of Amar-Halpert et al. (2017). Instead, we wanted to investigate to what extent similar effects of reactivation could be found in a paradigm that focuses on the learning of more complex visual objects. We decided to stay close to the object naming paradigm that has been used in several previous studies (Furmanski and Engel, 2000; Baeck and Op de Beeck, 2010; Baeck et al., 2014, 2016; Van Meel et al., 2016), rather than trying to come up with an object recognition task that would be as similar as possible to the texture discrimination task of Amar-Halpert et al. (2017). As a consequence, there are many differences between the two protocols on top of the difference in domain (texture vs objects), and that might affect the results given that perceptual learning is sensitive to a lot of variables, including stimulus parameters (Sagi, 2011). To mention a few differences, we used a different method to obtain thresholds (an adaptive procedure, in contrast to the method of constant stimuli), we found different threshold values (which could be because of the adaptive procedure, or to a different strength of masking), our paradigm included a preview of the stimuli which might facilitate performance before training, the stimulus-response mapping is more complex, the reactivation includes more trials because of having more stimuli, and we have a larger sample size which results in a higher power and thus a higher probability of finding group differences.

The improvements that we find because of learning are small in absolute magnitude, and so are the differences between groups. However, the differences are large in percentages, and with this paradigm we and others have consistently found highly replicable learning effects despite the small size in absolute terms (Furmanski and Engel, 2000; Baeck and Op de Beeck, 2010; Baeck et al., 2012). This object learning paradigm also results in very consistent and replicable effects. Nevertheless, we cannot exclude the possibility that our finding of less learning in the reactivation group would be related to specific properties of our methods, such as the way we estimate the thresholds or the limited temporal resolution with which we can adjust task difficulty (limited by the frame rate of the monitor).

The results of the current study support the claim that the previously noted improvements in the object naming task require large amounts of practice. In comparison to what was done in many previous studies (Furmanski and Engel, 2000; Baeck and Op de Beeck (2010); Baeck et al., 2012, 2014, 2016), the reactivation protocol is very short. It did not result in much additional improvement on top of the effect of the first day of extensive training. However, many experimental parameters might affect the strength of effects induced by reactivation, and we might simply not have found the optimal conditions. Thus, instead of concluding that reactivation has a smaller effect than the brute-force method of large amounts of practice, we hypothesize that its effects might simply depend more on the circumstances.

## Synthesis

Reviewing Editor: Li Li, New York University Shanghai Decisions are customarily a result of the Reviewing Editor and the peer reviewers coming together and discussing their recommendations until a consensus is reached. When revisions are invited, a fact-based synthesis statement explaining their decision and outlining what is needed to prepare a revision will be listed below. The following reviewer(s) agreed to reveal their identity: Sheng Li.

Both reviewers believe the ms has potential to be interesting. One reviewer raised questions regarding methodological issues that should be addressed before a recommendation can be made. I list their detailed comments below.

Reviewer 1

Review on manuscript titled "Perceptual learning with complex objects achieves in full-practice training, more than by short-reactivation strategy from memory reactivation". This pre-registered study focuses on a very timely topic. There are ongoing debates on the effectiveness of reactivation procedure and the boundary conditions of its underlying reconsolidation process. Amar-Halpert et al. (2017) showed surprising findings that brief reactivation could facilitate perceptual discrimination of simple textures. However, how such procedure could benefit the more complex object learning was not explored in the literature. The present study filled this gap by conducting a study with object learning that followed the general procedure of Amar-Halpert et al. (2017). The manuscript is clearly written and the methodology is properly adopted. The results provide an interesting comparison with Amar-Halpert et al. (2017)’s and could contribute significantly to the current debate. However, a couple of issues need to be clarified before the acceptance for publication.

1. The present study used near-threshold stimuli in reactivation trials, as in Amar-Halpert et al. (2017), to provide a fair comparison between the two studies. Given that the number of reactivation trials were small (5 per session), the attributes of the stimuli during reactivation thus become very critical. There were seemingly differences in the measurement of the threshold as well as the actual measured thresholds in the two studies. If this was indeed the case, the near-threshold stimuli in the two studies were not corresponding to the same performance level. The main conclusion of the present study is hinge on the equivalence of threshold values. I would suggest the authors to provide further information to clarify this issue and offer revised interpretation if the thresholds were different between the two studies.

2. What is the boundary of simple and complex visual processing that led to different findings of Amar-Halpert et al. (2017) and the present study? I can intuitive perceive that texture discrimination and object identification differ in terms of processing complexity/difficulty. However, it is not clear yet what kinds of results will be observed if we use a visual feature with middle complexity (e.g., a shape) in the learning/reactivation experiments.

3. In the results of learning specificity, it was not clear if the last two statistical tests were based on the day 5 data or the averaged data of day 1 and day 5. The method part did not mention these two tests. Also, they cannot be the main effects as no ANOVA was performed here (ANOVA could be a suitable examination here and in other places where multiple factors were involved).

4. Table 2 was not referred in the main text. So I was quite confused when I saw the letters (a, b, c, ...) after each statistical value, until Table 2 showed up at the end of the manuscript.

5. In the discussion, conflicting findings concerning the arguments around the idea of reconsolidation were listed. I would suggest to include Hardwicke et al. (2016) PNAS together with Walker and Stickgold (2016) as the former one was the original replication study.

Reviewer 2

In perceptual learning, human performance on visual tasks improves with practice. In most studies practice is intensive with hundreds of trial given on each training session. Amar-Halpert et al (2017) showed that one does not need to expose the trained subjects to so many trials but rather a few trials are sufficient after a standard (extensive) initial session of training. Amar-Halpert et al used the well-studied standard texture discrimination task, involving orientation based texture discrimination, considered to probe low-level visual processes. Here, in the present work, authors ask whether this magic of reactivation can be extended to an object naming task, assumed to probe recognition of complex visual stimuli. The results are indeed encouraging, with the naming task showing a behavior very similar to that of the texture task. Importantly, the two studies differ, not only in task but also in methodology, still showing similar results.

There are several serious issues with the present manuscript that need to be addressed by the authors before a recommendation can be made. I hope these issues can be fixed by re-analyzing the experimental results and a new write-up.

Major issues

1. The authors conclude in the abstract that "We found that traditional intensive training achieves a better improvement in object learning than reactivation-induced changes. The lack of benefit from reactivation suggests improvements in object recognition require large amounts of practice." This is strange, indeed there is more benefit from intensive training, in agreement with the previous study, but one cannot say that there is "lack of benefit from reactivation"! In fact, a comparison of the main results figures from the two studies shows a surprising similarity. The differences are minor, though here the small diff (∼3-4ms?) between the full-practice and the short-reactivation groups reaches statistical significance (larger sample?). These diffs definitely do not justify all the speculations developed by the authors, in particular given the technical limitations listed below (e.g. temporal resolution), please remove. The conclusions need to be reformulated.

2. The significance statement says: "We compare short reactivation and full-practice training and find that improvements in object recognition require large amounts of practice." This is a too general statement. You find improvement with short reactivation. You may argue not a sufficient one but this would be true only for the present paradigm (see limitations below) - a generalization to object recognition (assuming you measure object recognition) is not possible.

3. There are important methodological differences that need to be discussed. Here stimulus strength was manipulated by varying stimulus duration while in the texture task it is the "processing time" that is varied (ISI, time between stimulus and mask). Here threshold are measured using a staircase method, not in the texture task. Here reactivation includes more trials (25? vs 5?) and stimulus-response mapping is complex (5x5 vs 2x2). These are formalities that need to be stated when a "replication" is attempted, and there are several important practical issues related to these methodological aspects.

4. Varying stimulus duration affects the effective contrast of the target when using brief stimuli as done here. In fact, most probably the limiting factor for perception here is the stimulus contrast, not recognition or response mapping(?). It is possible that subjects improve their contrast sensitivity during practice, not their recognition ability.

5. Considering the above issue of stimulus duration, one needs to consider that the stimulus used on day 1 is of longer duration and higher effective contrast while after training duration and effective contrast are reduced. The reactivation group is exposed to stimuli with durations set according the thresholds obtained on the first day, thus longer and of higher contrast, while for the full-practice group this parameter is gradually reduced during practice. This is a problem since perceptual learning is specific to stimulus parameters (e.g. contrast; see Sagi 2011 for review), thus one expects less learning for the reactivation group.

6. The staircase method: here a 2down/1up adaptive method was used with stimulus duration increased or decreased by 10 ms (a limitation set by framerate). Given that threshold can go below 20ms (Fig 4), I don’t understand how such measurement method can produce reliable unbiased measurements. Please reanalyze the experimental results using percent correct recognition rates for each stimulus duration so that a psychometric curve can be obtained and thresholds be estimated by some standard fitting procedure.

7. The reactivation trials consisted of stimuli at threshold level estimated on day 1. How accurate the estimated individual thresholds are? Given the above mentioned limitations on threshold measurements probably not too accurate. Also stimuli can be presented with durations allowed by frame rate, so either too difficult or too easy, affecting learning (critical with the low thresholds obtained in the present study). Please provide thresholds values, stimuli durations and performance levels.

8. Preview: was a preview given by Amar-Halpert et al? Preview can affect learning and generalization (pre-training, introductory trials; see Sagi 2011 for review).

9. Learning rate: not sure this is a good term when comparing two points in time, rate implies some continuity. Related to that, the authors seem to assume that the right way to estimate rate is by normalizing the threshold differences due to learning to the initial threshold. This is OK if this ratio is invariant under threshold changes which, by looking at the available data, is most probably incorrect both here and in Amar-Halpert. Importantly, measurements on day 1 and 5 were based on different numbers of trials (N1 =2*N5), thus not clear if comparable (e.g. Censor et al, cited).

10. Discussion: Much of it needs to be removed, in particular the reference to fear conditioning is not of any relevance to the present work.

11. Regarding the difference in tasks, I am not sure it is that a significant diff considering that the task here is probably limited by stimulus contrast. The specificity measure could have helped here but not enough information is given considering the stimuli (i.e. the Fourier spectra seem to differ between the two stimuli groups used). Also note that learning is better generalized with short training sessions (Jeter et al, cited; see Sagi 2011 for review), thus finding generalization with the reactivation procedure is not of much surprise.

12. Final statement in discussion: "To be on the save side, we cannot conclude that in general a reactivation protocol will always fail to result in strong improvements of object learning. More research is needed. What we dare to conclude however, is that potential beneficial effects of reactivation in some circumstances would not be very universal, and that the best way to improve perceptual object processing is to practice a lot." ("save" probably means safe.) Again, not clear to me how such a strong statement can be made based on the current results and on the limited methodology employed. Please remove.

## Author Response

Synthesis of Reviews:

Significance Statement Comments for Author (Required):

n.a.

Comments on the Visual Abstract for Author (Required):

The visual abstract needs to convey the scientific message other than the text.

Synthesis Statement for Author (Required):

Both reviewers believe the ms has potential to be interesting. One reviewer raised questions regarding methodological issues that should be addressed before a recommendation can be made. I list their detailed comments below.

Authors’ response

We emphasize that this is a stage II registered report. The study design and analysis methods were approved in stage I. This has consequences for the review process at this stage, and for how we address the review comments. We keep following as much as possible the experimental and analysis plan that was approved in stage I, and provide further information as needed to address the review suggestions.

Reviewer 1

Review on manuscript titled "Perceptual learning with complex objects achieves in full-practice training, more than by short-reactivation strategy from memory reactivation". This pre-registered study focuses on a very timely topic. There are ongoing debates on the effectiveness of reactivation procedure and the boundary conditions of its underlying reconsolidation process. Amar-Halpert et al. (2017) showed surprising findings that brief reactivation could facilitate perceptual discrimination of simple textures. However, how such procedure could benefit the more complex object learning was not explored in the literature. The present study filled this gap by conducting a study with object learning that followed the general procedure of Amar-Halpert et al. (2017). The manuscript is clearly written and the methodology is properly adopted. The results provide an interesting comparison with Amar-Halpert et al. (2017)’s and could contribute significantly to the current debate. However, a couple of issues need to be clarified before the acceptance for publication.

Authors’ response

We thank the reviewer for the constructive evaluation of our study.

1. The present study used near-threshold stimuli in reactivation trials, as in Amar-Halpert et al. (2017), to provide a fair comparison between the two studies. Given that the number of reactivation trials were small (5 per session), the attributes of the stimuli during reactivation thus become very critical. There were seemingly differences in the measurement of the threshold as well as the actual measured thresholds in the two studies. If this was indeed the case, the near-threshold stimuli in the two studies were not corresponding to the same performance level. The main conclusion of the present study is hinge on the equivalence of threshold values. I would suggest the authors to provide further information to clarify this issue and offer revised interpretation if the thresholds were different between the two studies.

Authors’ response

For the original study design, and as approved at Stage I for this registered report, we decided not to do a close replication of the original study. Instead, we tested the generalization of the effect of reactivation to another domain of learning about more complex objects. We stayed very close to the design, the characteristics of perceptual learning (i.e. stimulus specificity), that has been used in many previous publications (Baeck et al., 2016, 2014, 2012; Baeck and Op de Beeck, 2010; Furmanski and Engel, 2000; Van Meel et al., 2016).

In the revised manuscript we emphasize this even more explicitly throughout the manuscript. We extended the discussion with more details on the various differences between our study and Amar-Halpert et al. (around line 408). Our point of view is that each of these changes could be relevant for explaining the discrepancy in results, but that from a theoretical point of view, in principle, the reactivation approach should generalize across many of these differences. That’s why we conclude that we need more research on the boundary conditions under which reactivation reaches its full potential (second to last sentence in the Discussion section).

To come back to the specific example of how thresholds were determined, Amar-Halpert et al. used a method of constant stimuli, while we used an adaptive procedure. Both methods are generally accepted as methods to determine thresholds, and our method was approved in the Stage I of this registered report. We agree that it is relevant to also mention this difference in the manuscript, which we now do.

2. What is the boundary of simple and complex visual processing that led to different findings of Amar-Halpert et al. (2017) and the present study? I can intuitive perceive that texture discrimination and object identification differ in terms of processing complexity/difficulty. However, it is not clear yet what kinds of results will be observed if we use a visual feature with middle complexity (e.g., a shape) in the learning/reactivation experiments.

Authors’ response

We agree that this boundary is difficult to define. We now mention this early-on in the Introduction, as follows: "While the boundary between simple and complex processing is difficult to draw, together these findings demonstrate that a wide variety of visual capabilities improve with extensive trainings."

3. In the results of learning specificity, it was not clear if the last two statistical tests were based on the day 5 data or the averaged data of day 1 and day 5. The method part did not mention these two tests. Also, they cannot be the main effects as no ANOVA was performed here (ANOVA could be a suitable examination here and in other places where multiple factors were involved).

Authors’ response

We now specify that these tests include the day 5 performance in the section on Specificity. ANOVA was not suggested in the context of the phase I approval, and it was also not done in the previous studies with the same paradigm. One complication with an ANOVA is that it would involve a design with at least 3 factors and interactions, while only a few comparisons are crucial for testing the hypotheses, and the predictions of these hypotheses do not simply map upon a main effect or interaction in the ANOVA.

4. Table 2 was not referred in the main text. So I was quite confused when I saw the letters (a, b, c, ...) after each statistical value, until Table 2 showed up at the end of the manuscript.

Authors’ response

We added the following text to the method section in line 236 on p11: "see Table 2"

5. In the discussion, conflicting findings concerning the arguments around the idea of reconsolidation were listed. I would suggest to include Hardwicke et al. (2016) PNAS together with Walker and Stickgold (2016) as the former one was the original replication study.

Authors’ response

We thank the reviewer for drawing our attention to include the original replication study. We have included Hardwicke et al. (2016) PNAS together with Walker and Stickgold (2016) on p 18.

-

Reviewer 2

In perceptual learning, human performance on visual tasks improves with practice. In most studies practice is intensive with hundreds of trial given on each training session. Amar-Halpert et al (2017) showed that one does not need to expose the trained subjects to so many trials but rather a few trials are sufficient after a standard (extensive) initial session of training. Amar-Halpert et al used the well-studied standard texture discrimination task, involving orientation based texture discrimination, considered to probe low-level visual processes. Here, in the present work, authors ask whether this magic of reactivation can be extended to an object naming task, assumed to probe recognition of complex visual stimuli. The results are indeed encouraging, with the naming task showing a behavior very similar to that of the texture task. Importantly, the two studies differ, not only in task but also in methodology, still showing similar results.

There are several serious issues with the present manuscript that need to be addressed by the authors before a recommendation can be made. I hope these issues can be fixed by re-analyzing the experimental results and a new write-up.

Authors’ response

We have reworded parts of the discussion and we have given more details on methodologic differences between studies.

Major issues

1. The authors conclude in the abstract that "We found that traditional intensive training achieves a better improvement in object learning than reactivation-induced changes. The lack of benefit from reactivation suggests improvements in object recognition require large amounts of practice." This is strange, indeed there is more benefit from intensive training, in agreement with the previous study, but one cannot say that there is "lack of benefit from reactivation"! In fact, a comparison of the main results figures from the two studies shows a surprising similarity. The differences are minor, though here the small diff (∼3-4ms?) between the full-practice and the short-reactivation groups reaches statistical significance (larger sample?). These diffs definitely do not justify all the speculations developed by the authors, in particular given the technical limitations listed below (e.g. temporal resolution), please remove. The conclusions need to be reformulated.

Authors’ response

We agree that our ability to find differences between the groups might be due to the larger number of participants. Finding this difference changes the conclusions with respect to Amar-Halpert. The current study has checked the statistic power and decided the expected number of subjects beforehand. The sample size is an important difference with the earlier study of Amar-Halpert. We have now added a discussion on the many differences between these studies around line 419, including sample size. In addition, we also emphasize more clearly in the revised manuscript that our study is not meant as a replication or refutation of Amar-Halpert’s findings, even though their study was obviously an inspiration and point of reference for our study.

It is indeed relevant to discuss the size of the group difference. The difference is small in absolute terms. We mention this in the Discussion as follows: "The improvements that we find due to learning are small in absolute magnitude, and so are the differences between groups. However, the differences are large in percentages, and with this paradigm we and others have consistently found highly replicable learning effects despite the small size in absolute terms (see e.g. Furmanski and Engel, 2000; Baeck and Op de Beeck, 2010; Baeck et al., 2012)." A further discussion of this point can be found below in the context of point 6.

2. The significance statement says: "We compare short reactivation and full-practice training and find that improvements in object recognition require large amounts of practice." This is a too general statement. You find improvement with short reactivation. You may argue not a sufficient one but this would be true only for the present paradigm (see limitations below) - a generalization to object recognition (assuming you measure object recognition) is not possible.

Authors’ response

We have reworded the significance statement to emphasize that we investigate a different domain and by ending with a more literal description of our findings rather than a general conclusion.

3. There are important methodological differences that need to be discussed. Here stimulus strength was manipulated by varying stimulus duration while in the texture task it is the "processing time" that is varied (ISI, time between stimulus and mask). Here threshold are measured using a staircase method, not in the texture task. Here reactivation includes more trials (25? vs 5?) and stimulus-response mapping is complex (5x5 vs 2x2). These are formalities that need to be stated when a "replication" is attempted, and there are several important practical issues related to these methodological aspects.

Authors’ response

We did not do a replication. It is even not a conceptual replication. We did not intend to claim we did, and we hope that we now avoid that impression even more. In the Discussion we now write explicitly around line 412: "It is important to note that our study was not meant to directly replicate the study of Amar-Halpert et al (2017). Instead, we wanted to investigate to what extent similar effects of reactivation could be found in a paradigm that focuses upon the learning of more complex visual objects."

In the same paragraph, we have added several sentences that draw the attention to the many differences between the studies, including the differences mentioned above. Note that these differences were approved in the Stage 1 of this registered report.

4. Varying stimulus duration affects the effective contrast of the target when using brief stimuli as done here. In fact, most probably the limiting factor for perception here is the stimulus contrast, not recognition or response mapping(?). It is possible that subjects improve their contrast sensitivity during practice, not their recognition ability.

Authors’ response

The approach that we have taken indeed involves a change in stimulus duration. We also did this in our earlier studies in this domain, it was approved in Stage I of this registered report, and we have borrowed this manipulation from the first studies with similar paradigms: Furmanski & Engel (2000, Vision Research) and Grill-Spector et al. (2000, Nature Neurosci.). Luckily that means that the underlying processes have been characterized quite well. In particular, Grill-spector et al demonstrated that the neural correlates of this training are found in object-selective cortex (LOC) and not in V1 where effects of stimulus contrast are typically found. This is further supported by effects of TDCS around area LOC. We have added the following paragraph to the Introduction to immediately refer the reader to the validation of this paradigm through results at the neural level: "This improvement in object recognition under perceptually challenging conditions seems related to activity in high-level object-selective cortex (Grill-Spector et al., 2000; Van Meel et al., 2016)."

5. Considering the above issue of stimulus duration, one needs to consider that the stimulus used on day 1 is of longer duration and higher effective contrast while after training duration and effective contrast are reduced. The reactivation group is exposed to stimuli with durations set according the thresholds obtained on the first day, thus longer and of higher contrast, while for the full-practice group this parameter is gradually reduced during practice. This is a problem since perceptual learning is specific to stimulus parameters (e.g. contrast; see Sagi 2011 for review), thus one expects less learning for the reactivation group.

Authors’ response

We have included the reference of Sagi (2011) in the new paragraph on the differences between studies (around line 423). The manipulation of stimulus duration together with stimulus-mask asynchrony as a measure to affect object recognition has been validated in prior research (see previous point), and was approved as such in the stage I approval of this registered report.

It is an interesting issue that the reactivation protocol does not adapt threshold values between days, while the hypothesis that reactivation changes learning predicts that the real threshold of subjects will improve over days as a consequence of this protocol. So the reactivation is probably no longer at threshold in the later days when the reactivation protocol has a large effect. This issue is most problematic when the reactivation has large effects, thus it is more a consideration for the study of Amar-Halpert than ours. So it unlikely explains why a study would have smaller effects of reactivation, and we think further speculation on this point would lead the discussion too much off-track from the main points.

6. The staircase method: here a 2down/1up adaptive method was used with stimulus duration increased or decreased by 10 ms (a limitation set by framerate). Given that threshold can go below 20ms (Fig 4), I don’t understand how such measurement method can produce reliable unbiased measurements. Please reanalyze the experimental results using percent correct recognition rates for each stimulus duration so that a psychometric curve can be obtained and thresholds be estimated by some standard fitting procedure.

Authors’ response

The staircase method is one of the standard methods for determining thresholds. It is impossible to reanalyze experimental results using percent correct recognition rates because this is not relevant in the case of the staircase method. The idea of a staircase method is to converge to a stimulus duration value (the threshold) that corresponds to a pre-set recognition performance. Fitting psychometric curves is also not relevant, because most trials are around the threshold value and thus the whole curve is not sampled to a sufficient degree. In a first publication with the used paradigm we have done some supplemental analyses with the psychometric fitting (supplemental material in Baeck and Op de Beeck, 2010). The results confirmed the findings obtained using the threshold values, but also illustrated the complications when trying to fit psychometric curves on data from an adaptive procedure. We now briefly mention this in the Methods around line 218 as follows: "Learning-related changes were quantified by the threshold values across sessions, which is the most obvious manner to characterize performance when using an adaptive procedure (psychometric curve fitting is difficult given the uneven sampling of this function, see Baeck and Op de Beeck, 2010)." Two other important reasons to not engage in psychometric curve fitting are that, first, the method and analysis of using the threshold values have been approved at stage 1, and, second, the method of using the threshold values is what we used for determining the stimulus durations in the reactivation protocol.

In every single study that we did, this approach has resulted in very clear learning effects, and many other effects have been found. Furthermore, as far as manipulations appeared again in different studies, such as the use of a control stimulus set to check object specificity, effects were always replicated. We agree that the temporal resolution of our manipulation of stimulus duration is not very fine-grained given the thresholds that are found, but with the number of trials and subjects that we typically include our methods are clearly sufficient to result in robust and replicable findings. A similar situation was observed in the first study with a related paradigm (Furmanski & Engel, 2000), with improvements of less than 10 ms from Day 1 to Day 5 using a 75 Hz monitor (which gives a temporal resolution of 13 ms). They had only 5 subjects and showed that such protocol can give very consistent learning curves at the level of individual participants, which is probably why we find such good replicability when using a similar paradigm with a larger sample size. We now mention this explicitly around line 431, as follows: "The improvements that we find due to learning are small in absolute magnitude, and so are the differences between groups. However, the differences are large in percentages, and with this paradigm we and others have consistently found highly replicable learning effects despite the small effect size in absolute terms (see e.g. Furmanski and Engel, 2000; Baeck and Op de Beeck, 2010). This object learning paradigm also results in very consistent and replicable effects. Nevertheless, we cannot exclude the possibility that our finding of less learning in the reactivation group would be related to specific properties of our methods, such as the way we estimate the thresholds or the limited temporal resolution with which we can adjust task difficulty (limited by the frame rate of the monitor)."

7. The reactivation trials consisted of stimuli at threshold level estimated on day 1. How accurate the estimated individual thresholds are? Given the above mentioned limitations on threshold measurements probably not too accurate. Also stimuli can be presented with durations allowed by frame rate, so either too difficult or too easy, affecting learning (critical with the low thresholds obtained in the present study). Please provide thresholds values, stimuli durations and performance levels.

Authors’ response

We agree that this information is very relevant. The threshold values can indeed not be derived from the current figure plots, as the plots show the thresholds across full sessions while the threshold/duration is determined based upon the last 4 staircases on day one and thus expected to be lower than for the full session. We now provide this additional information on page 14, as follows: "During the reactivation sessions, subjects were given brief training with the near-threshold trials. To estimate the individual threshold for each subject, the final four reversals of last four staircases on day one were averaged, mean {plus minus} standard error: 40.75 {plus minus} 1.86 ms. Based on this, the stimulus duration used for the reactivation trials was 41.54 {plus minus} 2.05 ms (note that per participant the duration is a multiple of 10). The average accuracy during the reactivation trials was 60 {plus minus} 3.86% on Day 2, 67.54 {plus minus} 4.03% on Day 3, and 70.62 {plus minus} 4.29% on Day 4."

8. Preview: was a preview given by Amar-Halpert et al? Preview can affect learning and generalization (pre-training, introductory trials; see Sagi 2011 for review).

Authors’ response

We have adhered to the standard object learning paradigm as we used it in many previous studies, and as it was approved in the Stage I of this report. We now mention that the preview is one of the differences with the study of Amar-Halpert et al. We agree that we cannot know to what extent it might affect the results of reactivation, which is why we conclude that more research is needed on the boundary conditions of this phenomenon.

9. Learning rate: not sure this is a good term when comparing two points in time, rate implies some continuity. Related to that, the authors seem to assume that the right way to estimate rate is by normalizing the threshold differences due to learning to the initial threshold. This is OK if this ratio is invariant under threshold changes which, by looking at the available data, is most probably incorrect both here and in Amar-Halpert. Importantly, measurements on day 1 and 5 were based on different numbers of trials (N1 =2*N5), thus not clear if comparable (e.g. Censor et al, cited).

Authors’ response

We agree with the reviewer that the term, learning rate, implies a continuous measurement. However, we would like to use a consistent terminology relative to the study of Amar-Halpert et’s study. We have added a statement to the paper (page 12) to explain the use of this term, as follows:" we would estimate the level of improvement. This measure of the training effect is sometimes referred to as a learning rate, which is a bit misleading as we do not have a continuous measurement. Here, to use a consistent terminology relative to the study of Amar-Halpert et al., we also use the term learning rate."

10. Discussion: Much of it needs to be removed, in particular the reference to fear conditioning is not of any relevance to the present work.

Authors’ response

We have reworded the fear conditioning part to make it clearer, and similar for the brief reference to the motor learning domain. This reference is relevant because it illustrates that uncertainties about reactivation learning and its boundary conditions are not limited to the domain of perceptual learning. Note that we phrase everything in terms of "a lot is unknown" and not in terms of "findings are not replicable".

11. Regarding the difference in tasks, I am not sure it is that a significant diff considering that the task here is probably limited by stimulus contrast. The specificity measure could have helped here but not enough information is given considering the stimuli (i.e. the Fourier spectra seem to differ between the two stimuli groups used). Also note that learning is better generalized with short training sessions (Jeter et al, cited; see Sagi 2011 for review), thus finding generalization with the reactivation procedure is not of much surprise.

Authors’ response

The previous points have already addressed the issue of stimulus contrast and we have added information to the manuscript about studies that validated that learning in this paradigm involves object recognition (e.g. Grill-Spector et al., 2000; Van Meel et al., 2016). The Fourier spectra are indeed different between the different individual stimuli. One of the earlier studies with the object learning paradigm (Baeck & Op de Beeck, 2010) involved stimuli matched for the Fourier spectrum because one of the goals of that study was a comparison between backward masking and the addition of Fourier noise. The object specificity in that earlier study was very similar to what we find in the current study.

It is misleading to say that our findings show generalization with the reactivation procedure. Generalization suggests that there is more learning after reactivation (learning for both the trained stimuli and for the untrained stimuli), while the opposite true: after reactivation we find less learning for the trained stimuli and then it is not a surprise that we find less of a difference with the control stimuli.

12. Final statement in discussion: "To be on the save side, we cannot conclude that in general a reactivation protocol will always fail to result in strong improvements of object learning. More research is needed. What we dare to conclude however, is that potential beneficial effects of reactivation in some circumstances would not be very universal, and that the best way to improve perceptual object processing is to practice a lot." ("save" probably means safe.) Again, not clear to me how such a strong statement can be made based on the current results and on the limited methodology employed. Please remove.

Authors’ response

We have deleted it, and now we conclude: "However, many experimental parameters might affect the strength of effects induced by reactivation, and we might simply not have found the optimal conditions. Thus instead of concluding that reactivation has a smaller effect than the brute-force method of large amounts of practice, we hypothesize that its effects might simply depend more upon the circumstances." Also in the significance statement we have toned down the conclusions.
